# Artificial Intelligence-Based Quality Assessment of Histopathology Whole-Slide Images within a Clinical Workflow: Assessment of ‘PathProfiler’ in a Diagnostic Pathology Setting

**DOI:** 10.3390/diagnostics14100990

**Published:** 2024-05-09

**Authors:** Lisa Browning, Christine Jesus, Stefano Malacrino, Yue Guan, Kieron White, Alison Puddle, Nasullah Khalid Alham, Maryam Haghighat, Richard Colling, Jacqueline Birks, Jens Rittscher, Clare Verrill

**Affiliations:** 1 Department of Cellular Pathology, John Radcliffe Hospital, Oxford University Hospitals NHS Foundation Trust, Oxford OX3 9DU, UK; 2 Nuffield Department of Surgical Sciences, University of Oxford, Oxford OX3 9DU, UK; 3 Department of Cellular Pathology, Royal Berkshire Hospital, Royal Berkshire NHS Foundation Trust, Reading RG1 5AN, UK; 4 Department of Engineering Science, University of Oxford, Oxford OX1 3PJ, UK; 5 School of Electrical Engineering and Robotics, Queensland University of Technology, Brisbane, QLD 4000, Australia; 6 Nuffield Department of Orthopaedics, Rheumatology and Musculoskeletal Sciences, University of Oxford, Oxford OX3 7LD, UK; 7 NIHR Oxford Biomedical Research Centre, Oxford University Hospitals NHS Foundation Trust, Oxford OX3 9DU, UK

**Keywords:** digital pathology, whole slide images, artificial intelligence, quality control, automation, focus quality, histopathology, diagnosis

## Abstract

Digital pathology continues to gain momentum, with the promise of artificial intelligence to aid diagnosis and for assessment of features which may impact prognosis and clinical management. Successful adoption of these technologies depends upon the quality of digitised whole-slide images (WSI); however, current quality control largely depends upon manual assessment, which is inefficient and subjective. We previously developed PathProfiler, an automated image quality assessment tool, and in this feasibility study we investigate its potential for incorporation into a diagnostic clinical pathology setting in real-time. A total of 1254 genitourinary WSI were analysed by PathProfiler. PathProfiler was developed and trained on prostate tissue and, of the prostate biopsy WSI, representing 46% of the WSI analysed, 4.5% were flagged as potentially being of suboptimal quality for diagnosis. All had concordant subjective issues, mainly focus-related, 54% severe enough to warrant remedial action which resulted in improved image quality. PathProfiler was less reliable in assessment of non-prostate surgical resection-type cases, on which it had not been trained. PathProfiler shows potential for incorporation into a digitised clinical pathology workflow, with opportunity for image quality improvement. Whilst its reliability in the current form appears greatest for assessment of prostate specimens, other specimen types, particularly biopsies, also showed benefit.

## 1. Introduction

The implementation of digital pathology (DP) within the diagnostic setting has gained momentum in recent years, and the promise of artificial intelligence (AI) is now becoming reality to aid the pathologist in diagnosis and in the assessment of features that may guide management and predict prognosis. However, there remain challenges within the real-world setting in the assurance of quality of whole slide images (WSI). Quality of WSI of histopathology slides is recognised as a key factor in the acceptability of DP to practising histopathologists. Accurate diagnosis is reliant upon the histological features seen, and whilst there are recognised ‘pitfalls’ in digital diagnosis of which a pathologist must be aware [1,2,3], the WSI quality must not be inferior to glass slides (GS) for making clinical diagnoses.

Quality issues are also recognised to impact on the development and performance of AI tools for histopathology [4,5,6,7,8], and without assurance of performance reproducibility of such tools, their approval by regulatory bodies and their integration into the clinical setting will remain limited.

**Figure 1:**
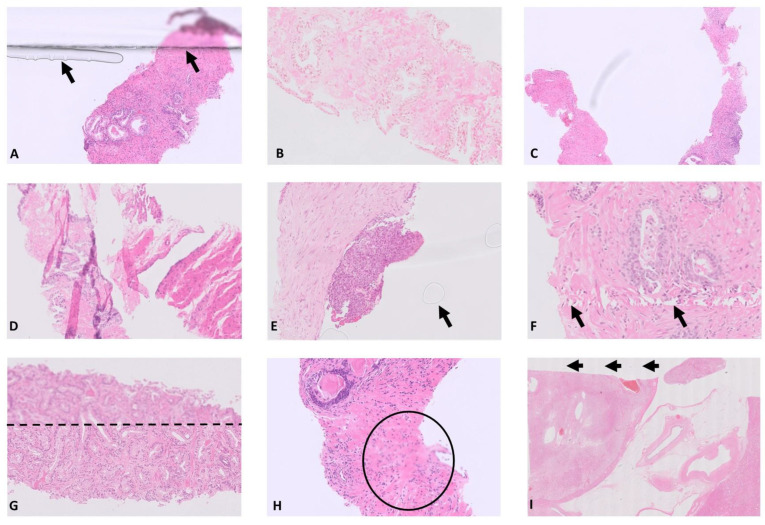
(**A**–**F**) Examples of quality-impacting features which may be introduced during the production of a glass slide. These may all impact on digital image quality when the slide is scanned, commonly causing problems with focus due to an uneven tissue section or physical artefact. (**A**) Tissue under the coverslip of the slide with uneven mountant obscuring some of the detail of the edge of the tissue section (arrowheads). (**B**) Pale H&E stain with lack of contrast. (**C**) Several focal areas of dirt under the coverslip (black marks). (**D**) Folded areas of tissue causing uneven section thickness and impacting on focus of the image. (**E**) Bubbles under the coverslip (arrowhead). (**F**) Tissue ‘scoring’ (arrowheads) which may, for example, be due to calcified material in the section. (**G**–**I**) Examples of poor-quality focus in a digitised image of a glass H&E slide (WSI). (**G**) ‘Stripe’ artefact with a clearly out-of-focus area in the upper image defined by the broken horizontal line. This can be multifactorial. (**H**) Small out-of-focus areas (circle) which are seen at high power only and therefore difficult to detect on a quick ‘screen’ of the WSI after scanning; these are usually detected by the pathologist at the time of the diagnostic read. (**I**) ‘Venetian blind’ artefact commonly due to contaminant on the camera lens, such as mountant. This results in evenly distributed vertical stripes across the image, example stripes seen here are indicated by the arrow heads. This is the least common of the focus-related issues encountered but invariably requires re-scan of the slide before the WSI is suitable for diagnosis. WSI = whole slide image.

The importance of quality control for WSI is emphasised within the literature encompassing the transition to DP [9,10,11,12]. Quality can be impacted at all stages of the production of a histology glass slide by both tissue-specific features and laboratory processing features [13], resulting in ‘pre-analytical’ artefacts familiar to the diagnostic pathologist (Figure 1A–F). Whilst some features can be accommodated by the experienced pathologist reading GS on the light microscope and have negligible diagnostic impact (such as air bubbles under the coverslip and areas of tissue folding), these features can impact significantly on the quality of the digitised image of the slide, potentially impacting its diagnostic suitability. Significant quality-impacting features can also be introduced at the scanning stage (Figure 1G–I). Within the digitised pathology workflow, therefore, a means to identify and intercept poor quality images prior to their ‘release’ to a pathologist for diagnostic interpretation should be considered in order to reduce the potential for impact on the production of the diagnostic report, and also the future potential for impact on AI outputs.

Quality should be considered at all stages during the production of a GS, during the scanning process itself, and prior to release of an image to the pathologist for diagnosis. Good laboratory practices are necessary to minimise ‘pre-analytical’ artefacts, and to ensure, for example, the correct placement of the tissue on the slide. Additional considerations important specifically for the digitisation process include the positioning of the slide label and cleaning of the GS, including removal of ink marks. The importance of these processes is highlighted in a recent report of a Quality Management System in DP operations in a clinical setting [14], which outlines a five-step QC process covering the preanalytical and analytical phases of WSI. 

Significantly, current DP set-ups on the whole do not have automated QC in their workflow. Whilst there are QC elements incorporated into digital scanners, these are generally considered insufficient to detect all possible quality issues with the scanned WSI, and as a result this task is reliant upon the manual inspection of scanned images, typically by trained laboratory staff. This is emphasised in the report from Ardon et al. [14] who suggest that review of WSI by their dedicated digital scan team requires one full time employee for every three to four high-throughput scanners, which is aspirational and may not be possible in many laboratories. Furthermore, manual QC of WSI is error-prone, with interpersonal variation in quality assessment [15]. Whilst issues affecting large areas of an image are relatively easy to identify, substantial time is required to screen a digital image for more focal and potentially subtle issues that may impact usability, and these are therefore often undetected until an image is scrutinised during the diagnostic read.

AI offers the potential for automation of some of the QC operations within pre-analytical stages which can reduce manual labour requirements including sample tracking and assessment of completeness of tissue sections [10,16]. Its utility for the QC of WSI following the digitisation process has been suggested; however, to date, this is not routine within clinical pathology laboratories due to lack of solutions which can assess the range of real-world artefacts encountered and provide a user-friendly output. Whilst automated QC tools exist for digitised pathology WSI [5,8,10,17], these are variably suited to be integrated into a real-time clinical workflow. Most are specifically designed to detect or modify a single quality issue such as focus quality [18] or image sharpness [19], whilst other tools exist to aid the ‘normalisation’ of colour in WSI [5,20]. As such, these tools are more suited to the research setting, potentially in the development or validation of AI tools, rather than the clinical workflow where, in reality, a wide range of quality issues exist. Automated QC tools with the ability to assess a more diverse range of artefacts [21] are limited in current practice, with none being employed routinely within a clinical setting.

We previously introduced PathProfiler [22] which is an open-source AI tool for the automated quality assessment of histopathology WSI. PathProfiler was developed through a collaborative effort between software engineers and diagnostic histopathologists to investigate the quality of WSI, initially within a historic cohort of prostate specimens. This AI solution automates the process of quality control of digitised images to predict their usability at the diagnostic level, and through the prediction of the range of artefacts present it can provide an indication as to whether an intervention such as re-scanning or re-staining may be beneficial. Our experience with PathProfiler in the automated analysis of image quality in the research setting highlighted its potential to aid improvement in WSI quality within a diagnostic workflow. In this study, we therefore sought to investigate the utility of PathProfiler within a digitised real-world clinical histopathology workflow, in real time, specifically to determine the feasibility of incorporating PathProfiler into the workflow to identify WSI of suboptimal quality for diagnosis, to evaluate its quality assessment (QA), and to establish the potential impact of PathProfiler within the clinical setting.

PathProfiler was trained and validated on a cohort of WSI of prostate specimens, predominantly prostate biopsies. Accordingly, the cohort of the routine workload selected to be analysed during this study (external referrals excluded) was genitourinary (GU) pathology cases. In our tertiary referral centre, around one-third of the current GU workload is prostate specimens (with prostate biopsies representing around 23% of the annual GU workload); however, the case mix includes the range of tissue types expected within the specialty. Acknowledgment is needed at the outset that, prior to the study, the performance of PathProfiler in relation to QA of non-prostate GU specimens was relatively unknown.

## 2. Materials and Methods

### 2.1. PathProfiler Quality Assessment Functionality and Output

PathProfiler provides a prediction of the usability of a WSI at slide level based upon overall image quality, as well as a prediction of the focus and staining quality. This is presented as a score for usability, the ‘Usability Score’ (US) of 0–1, and for the focus and staining quality of 0–10 (for full details see [22]). 

PathProfiler operates initially at patch level to provide an assessment of the presence of artefacts and the image quality, then these predictions are mapped to a slide-level scoring system. In brief, tissue regions are extracted by a tissue segmentation model and divided in a grid of patches: for each of these patches, PathProfiler predicts a set of quality measures, which include the quality of focus and H&E staining and the presence of specific artefacts recognised to impact on image quality, which include tissue folding, poor staining, and other miscellaneous features (such as dirt and ink). A score is generated for the H&E staining quality and for the focus quality on a continuum between 0 and 1, which matches subjective correlation at 0 (no quality issue), 0.5 (slight quality issue), and 1 (severe quality issue). These predicted scores at patch level are then aggregated to generate predicted usability, focus, and staining scores at the slide level, which would correlate with subjective assessment of the same WSI by a pathologist. Whilst no slide-level scores are assigned to ‘tissue folding’ and ‘other’ artefacts, heatmaps generated by PathProfiler provide an indication of where such artefacts might be located in an image.

For the purpose of this current feasibility study, for ease of interpretation of the PathProfiler output and to therefore enhance usability in the clinical setting, we limited the output to the overall usability score, as this was found to be highly predictive of overall image quality in the previous study [22]. The overall usability score outputted by the model is a real number between 0 and 1. A cut-off threshold for diagnostic purposes has been set at 0.4, mapping the score to a binary value, i.e., 0 (unusable) or 1 (usable). However, this cut-off score can be altered to align with user or laboratory preference.

We included scores for H&E staining quality and focus issues in the information provided to the Biomedical Scientist (BMS) team and pathologist to determine whether, in a real-world setting, this was helpful in indicating the ‘source’ of the suboptimal WSI quality. The predicted quality overlays produced by PathProfiler, or ‘heatmaps’, which were automatically generated to provide an indication of where a particular artefact might be present, were not available in real time within the study setup but were also made available for interest with the intention of assessing their utility in a real-world setting as an adjunct to the available quality scores.

### 2.2. Study Setting

This study took place within a fully digitised Cellular Pathology laboratory which has accreditation for the use of DP under the United Kingdom Accreditation Service (UKAS) ISO:15189 (Medical Laboratory Accreditation—ISO 15189 (ukas.com, accessed on 30 April 2024), operating a digital pathology set up (Philips Image Management System, Philips Ultrafast Scanner version 3) since 2018, with full digitisation of our surgical pathology workload since 2020 [3,23]. The installed DP solution has no fully automated QC process and currently our QC pathway for WSI relies upon our time-pressured trained laboratory staff and is thus limited to a simple check of the scanned image for major issues flagged by the scanner and block-checking WSI prior to release of the image on the Information Management System (IMS) to the pathologist for diagnosis. Therefore, almost all quality issues currently remain undetected until the diagnostic read, at which time the pathologist can defer to GS for reporting or request a re-scan.

In terms of the pre-analytical laboratory practices prior to slide scanning, the handling of the surgical specimens within the laboratory is in line with standard practice, and with specific specimen dataset guidance from the Royal College of Pathologists as applicable [24], which is incorporated into laboratory specific Standard Operating Procedure (SOP) documents for each specialty. Our laboratory is UKAS accredited (registration number 8415) and we undergo regular inspection for conformance with internationally recognised standards of ISO 15189:2012 [medical laboratory accreditation]. In brief, biopsy specimens less than 5 mm require a minimum of 2 h to fix in formalin and are embedded and processed overnight or (if urgent) via a 4-h schedule. For resection specimens, the formalin fixation time is usually overnight or up to 24 h to ensure adequate fixation, based upon the specimen type (adhering to published guidance), and to an extent also on an individual case basis as determined by the pathologist. In our centre, the biopsy and ‘chippings’ cases (such as bladder tumours and prostate chippings) are handled by Health and Care Professions Council (HCPC)-registered Biomedical Scientists, who also handle radical prostatectomy specimens. Other surgical resection specimens for GU cases are handled and cut-up by the GU pathologist team, or one of the trainee pathologists under supervision. Embedding and sectioning of specimen blocks in the lab is done in accordance with UKAS-accredited standardised laboratory protocols.

Regular assessments of glass slide quality are conducted through the UK national external quality assurance schemes (UKNEQAS), which runs 6 assessments over a 12-month period to ensure our slides are comparable and valid. Internal quality assurances are also conducted within the lab to ensure slide quality, such as regular manual checks by a HCPC-registered Biomedical Scientist to check they meet the set minimum acceptance criteria.

This study was conducted as a quality improvement exercise with approval of our local departmental clinical governance group, and as such did not require formal ethics approval. The study was carried out with a view to routine implementation if successful.

Feedback was collated regularly from the study team informally during the study, and following the study, anonymised formal fesaedback was sought via the online SurveyMonkey survey tool (www.surveymonkey.com, accessed on 10 April 2024).

### 2.3. Integration of PathProfiler into the Clinical Workflow

PathProfiler was incorporated into the digital pathology workflow within the lab and ran in real time within the clinical workflow for a 3-week period from Monday 27 February to end of Sunday 19 March 2023.

**Figure 2:**
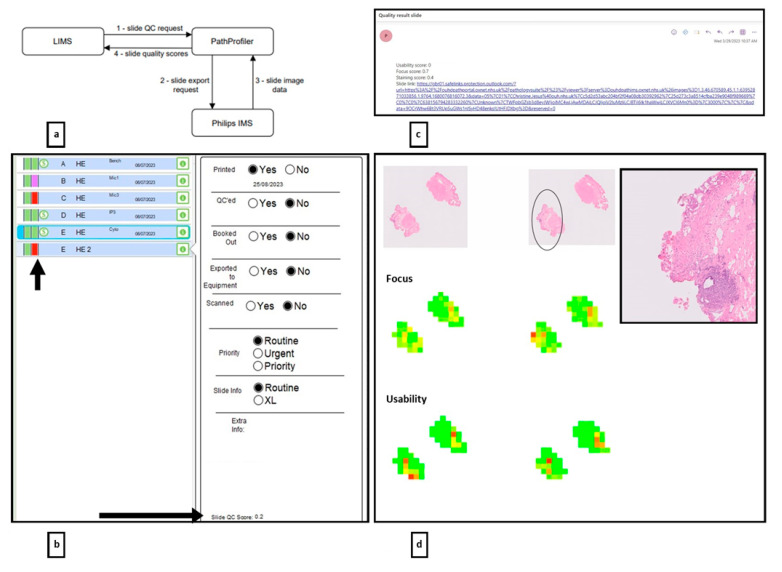
PathProfiler was integrated into the real time clinical workflow as shown in (**a**). The output of PathProfiler to flag WSI requiring attention due to suboptimal US (0.4 or less) was via integration of the US into the LIMS and by concurrent automated email to the Biomedical Scientist. Following the PathProfiler analysis of each WSI, a coloured flag was displayed for each slide on the LIMS (short arrow, (**b**)). Those WSI with suboptimal US usability scores had a red flag which would highlight the image to the Biomedical Scientist. A purple flag indicated WSI of appropriate quality (US = 0.5 or above). The actual US for each WSI was also available within the LIMS within the Information tab for each slide (long arrow). When a slide was re-scanned, the flags and US would be refreshed and only scores for the re-scanned WSI would be on display on the LIMS. (**c**) is an example of an automated email to the Biomedical Scientist team to alert them to a WSI which required attention. The US is detailed in the email together with the slide details and a direct link to the WSI on the IMS. (**d**) is an example of a quality overlay or ‘heatmap’ generated by PathProfiler to indicate visually the areas of a WSI which are predicted to be of lower quality (as determined by the US), and to indicate the predicted focus and H&E staining quality. This example is of a bladder biopsy with an area of predicted low US (red on heatmap) corresponding to a lower focus quality score (red on ‘focus quality’ heatmap). Subjectively, in this region of the H&E (top of image) there is an area of poor focus quality (inset H&E) which corresponds to the area on the heatmap. WSI = whole slide image, US = usability score, LIMS = laboratory management system, IMS = information management system.

For each scanned slide with a GU case code, the Laboratory Information System (LIS) automatically sent a QC request to the server hosting PathProfiler, at which point the corresponding slide image was exported from the Philips IMS and analysed by PathProfiler. The produced quality scores were then returned to the LIS (Figure 2a). All the components in the workflow were hosted on our internal trust IT systems behind our institutional firewall.

WSI with a US of 0.4 or less were flagged to the Biomedical Scientist (BMS) team for manual review (Figure 2b,c). Quality overlays (heatmaps) to indicate areas of a WSI which required visual inspection/attention were emailed separately to the study team at the end of each day to assess whether these would be a beneficial component of the PathProfiler output to incorporate in real time (Figure 2d).

**Figure 3:**
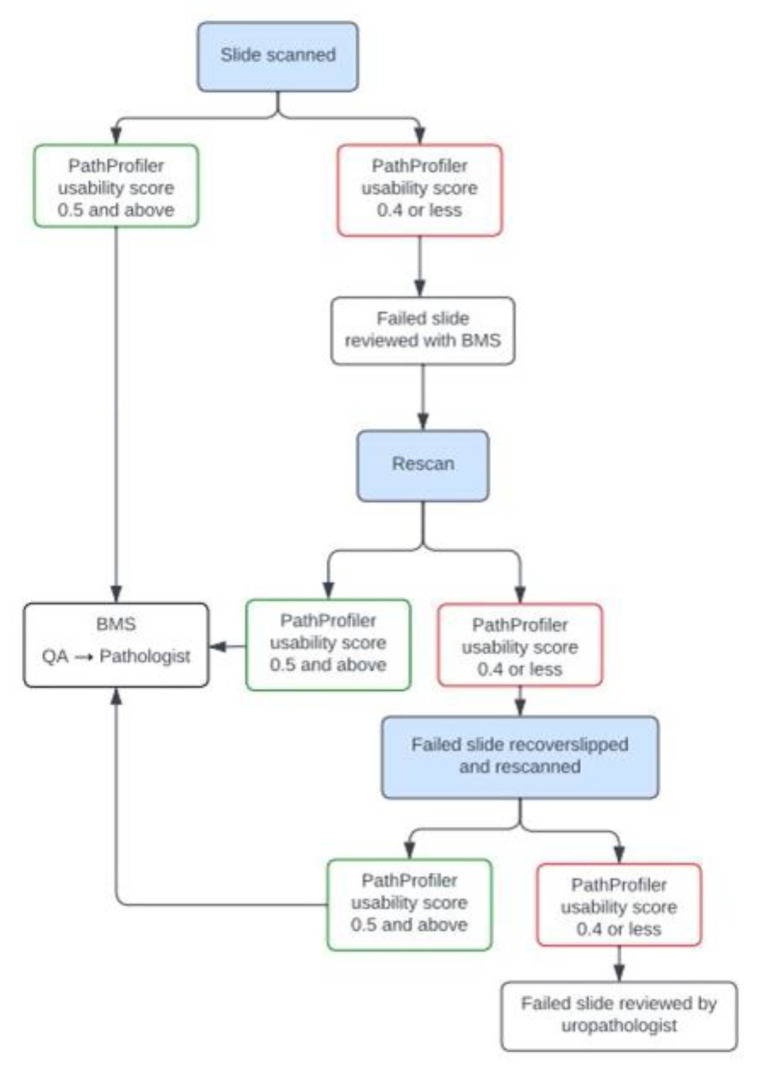
Pathway for digitisation of H&E slides with integration of PathProfiler within the workflow. BMS = biomedical scientist, QA = quality assessment.

Flagged slides were manually assessed for obvious quality-impacting features, and then cleaned, re-scanned and re-analysed by PathProfiler and flagged again if the US was 0.4 or less. WSI flagged for a second time were manually assessed to determine the requirement for any further remedial action (see Figure 3 for the pathway).

### 2.4. Comparative Subjective Assessment of PathProfiler Performance 

Each flagged WSI was subjectively assessed by a specialist GU pathologist with over 3 years DP experience. The assessment was made in accordance with the level of scrutiny of the WSI needed for diagnosis, which was tissue- and specimen-specific; for example, prostate biopsies required the highest level of focus quality and at high power, whereas selected slides from a larger resection specimen might require review only at low power for diagnostic purposes.

**Figure 4:**
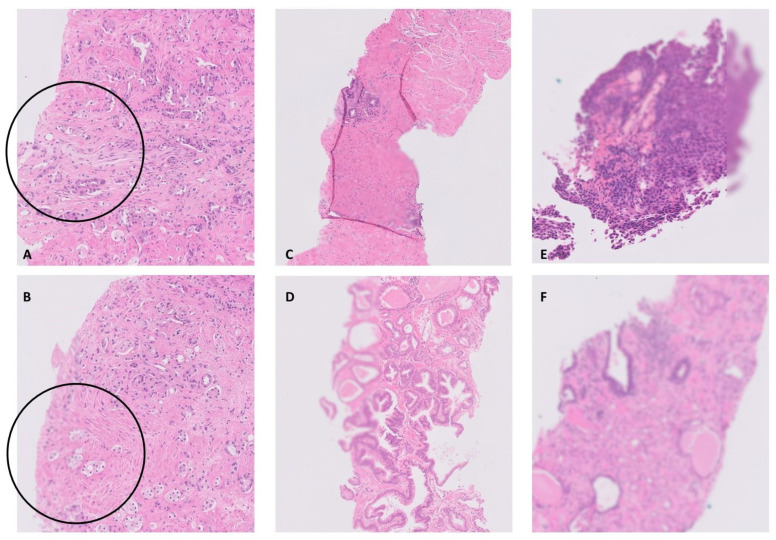
Subjective assessment of image quality was at the level required for diagnostic purposes. In the case of prostate biopsies, the image quality needed to be high and at high power magnification. Quality-impacting issues were categorised as no significant issue/minimal issue (not impacting on diagnosis), minor issue (may not impact on diagnosis), and severe issue (impacting on diagnostic usability). The examples in (**A**,**B**) show very minor out-of-focus areas (circled) which would not impact on diagnosis (no significant issue). (**C**,**D**) show ‘minor’ issues where the focus quality is suboptimal but the detail of the image is probably sufficient for diagnostic assessment, whereas in (**E**,**F**) the quality of focus is insufficient for diagnosis (severe issue).

The quality issues were categorised as (i) no significant issue/minimal issue (no impact on diagnostic usability), (ii) minor issue (may not impact on diagnostic usability), (iii) severe issue (impacting on diagnostic usability), see Figure 4. The tissue in the WSI was assessed equally for this purpose, regardless of its importance for diagnosis.

### 2.5. Assessment of the Impact of PathProfiler on the Turnaround Time of Diagnostic Cases

The workflow impact was assessed in relation to the overall turnaround time (TAT), defined as time from clinical sample receipt in the lab to authorisation of the diagnostic report, and separately the laboratory TAT (sample receipt to ‘release’ of case to pathologist) and the pathologist reporting TAT (time case ‘released’ from lab for reporting to authorisation of the report). TAT was reviewed for PathProfiler-analysed cases during the study period, and for a random comparable 3-week period preceding and after the study, and was limited to prostate biopsy cases given the close monitoring of their TAT and therefore a need to understand any potential negative impact on TAT encountered as a result of incorporation of PathProfiler. Cases were not analysed by PathProfiler in either of these separate 3-week periods. A sub-analysis of specific TATs for the prostate biopsy cases with flagged WSI was also performed.

## 3. Results

A total of 1254 consecutive GU WSI were analysed by PathProfiler from 184 GU cases. The case mix (see Table 1) included 82 prostate tissue specimens and 32 GU excision/resection cases (UREs). Of the total 1254 WSI, 46% (574/1254) were prostate biopsies (from 61 cases).

### 3.1. Summary of PathProfiler-Flagged WSI

**Figure 5:**
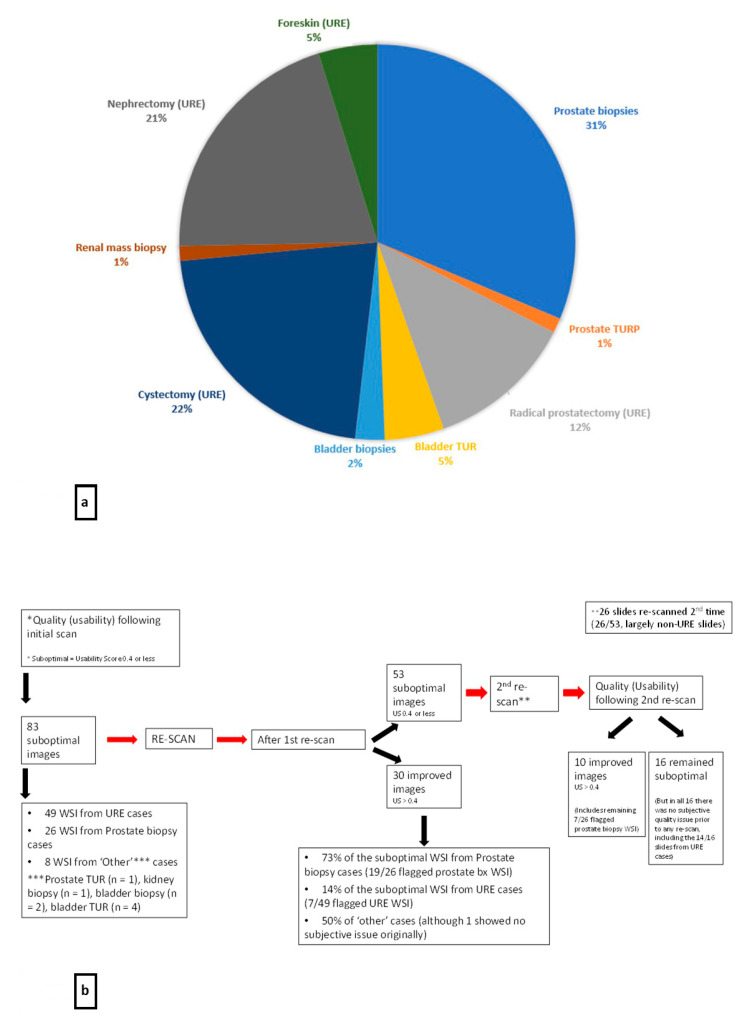
Summary of PathProfiler output. (**a**) shows the distribution of the 83 flagged Genitourinary WSI with PathProfiler US 0.4 or less (defined cut-off for suboptimal quality). (**b**) summarises the outcome of the Quality assessment by PathProfiler for the WSI during the study. Unless specified, the assessment in this figure is not related to the subjective assessment of the WSI. Note that not all of the slides were re-scanned a second time, based upon the early observation that the flagged URE slides were unlikely to have corresponding suboptimal quality on subjective assessment, and hence re-scanning was unlikely to be of benefit. WSI = whole slide image, US = usability score, TURP = transurethral resection of prostate tissue, TUR = transurethral resection specimen (usually of bladder tumour), URE = urology excision/GU resection specimen.

Of 1254 WSI scanned by PathProfiler, 83 had a US of 0.4 or less (6.6% of the WSI), representing slides from 28 separate cases (15%, 28/184 of cases). Of the total flagged WSI, 49 (59%) were from GU resection cases (URE), but excluding these, overall 2.7% of the WSI (34/1254) were flagged. The distribution of flagged WSI across the GU cases is shown in Figure 5a.

Following re-scan of slides where the WSI had been flagged as ‘suboptimal’, PathProfiler analysis showed improvement in the US for 30 WSI (36%, 30/83 slides), including 73% of the prostate biopsy slides (19/26 flagged prostate biopsy WSI). The second re-scan resulted in improvement of the US for the WSI for a further 10/26 re-scanned slides, including the remaining seven flagged prostate biopsy WSI. Remedial action therefore resulted in improved US for 100% of the flagged prostate biopsies. See Figure 5b for an overview of the PathProfiler output and actions during the study period.

### 3.2. Comparative Subjective Assessment of PathProfiler Output 

Of the initially flagged 83 WSI, pathologist assessment showed no significant/minimal issue in 49% of WSI (41/83), of which 95% (39/41) were from URE. A minor quality issue was seen in 31% of WSI (26/83), and 19% of the WSI (16/83) showed a severe quality issue and would require remedial action prior to the WSI being suitable for diagnostic use. Overall, none of the suboptimally scored WSI from the URE cases subjectively showed severe quality issues, but when UREs were excluded, the concordance between the US and there being a diagnostically significant issue on subjective quality assessment was improved (Table 2).

**Figure 6:**
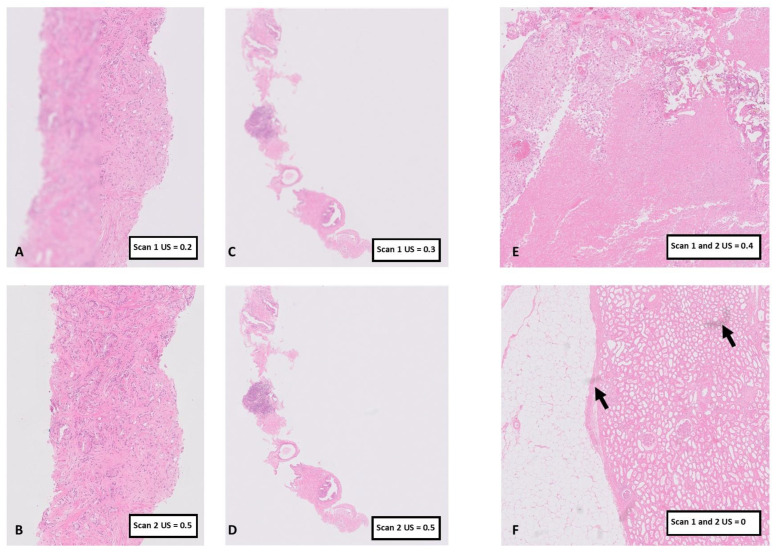
Examples of cases flagged by PathProfiler with US 0.4 or less and the outcome of re-scan. In (**A**) there is a clear ‘stripe’ artefact in this prostate biopsy causing a severe focus issue with US of 0.2, but on re-scan shown in (**B**), the US improved to 0.5 with subjective improvement in quality. The prostate biopsy in (**C**) shows a severe focus issue, which was subjectively improved on re-scan (**D**) which was also reflected in the second US of 0.5. (**E**,**F**) are examples of urology resection cases where the US did not reliably correlate with the subjective assessment. In (**E**) the bladder TUR shows areas of necrosis and diathermy resulting in pale ‘featureless’ areas on the H&E, which may have impacted on PathProfiler assessment. The original US for this WSI was 0.4 and this did not improve following re-scan, but subjectively there was no true issue with image quality. (**F**) is an example of a kidney resection where the US was 0 before and after re-scan. Subjectively, there was no significant issue with image quality—there were small foci of wax (dark areas, arrows) with minimal focus issues. US = Usability Score, TUR = transurethral resection, WSI = whole slide image.

The 83 slides from flagged WSI were cleaned and re-scanned. Of these new WSI, 30 (36%, 30/83) had improved US of 0.5 or above, which was concordant with subjective improvement in 24 WSI (80%, 24/30, examples shown in Figure 6A–D). It is noteworthy that five of the remaining 6/30 suboptimal WSI did not have a significant image quality issue on initial subjective assessment (prior to re-scan, examples shown in Figure 6E,F), four of these five were from UREs, and the other was from a bladder TUR showing severe cautery artefact and inflammatory exudate considered likely responsible for the low US rather than there being an issue with the digital image quality per se (Figure 6E). The remaining one WSI (also a URE slide) was considered to have a quality-impacting issue on subjective assessment that remained following re-scan in spite of the improved US, although this issue was considered to be minor and would not have impacted on diagnosis.

Of the 53 persistently suboptimal WSI flagged a second time, 26 slides were re-scanned a second time, some following replacement of the coverslip. Of these 26 re-scanned slides, the new images had improved US scores to >0.4 for 10 slides (38%, 10/26), including the seven remaining flagged prostate biopsy WSI, and with concordant improvement in subjective assessment. Of the remaining 16 WSI, none were considered to have a significant quality issue in need of further action on subjective assessment.

### 3.3. Quality Assessment of WSI from Prostate Biopsy Cases

PathProfiler was developed using prostate tissue specimens, and it was therefore of interest to sub-analyse the output for these cases, focussing on the prostate biopsies, given the relative discordance of the comparative AI and subjective analysis of URE cases as previously discussed.

Of the 574 analysed prostate biopsy WSI, 26 were flagged by PathProfiler (4.5%, 26/574), which were from 14 separate cases. On subjective assessment, almost invariably the quality issue was related to focus, typically small areas with out-of-focus patches or stripe artefacts, not all being diagnostically impactful. Subjective assessment at a level of quality required for diagnosis revealed that of the 26 flagged slides, three showed very minor (non-significant) issues (11%), nine showed minor issues (35%), and 14 showed severe issues (54%). The comparative mean PathProfiler US score for WSI within these three categories was 0.37, 0.34, and 0.2, respectively.

Of the 26 flagged prostate biopsy WSI, 19 (73%) saw an improvement in US following cleaning and re-scanning of the slide (Figure 6A,B), with 100% improvement in US to 0.5 or above following the second re-scan, with some of these slides having also been re-coverslipped. Subjective improvement in image quality was confirmed in all 26 re-scanned WSI, although in two WSI there remained minor focus quality issues (not impactful in terms of diagnosis).

### 3.4. Impact of PathProfiler on Diagnostic Turnaround Times of Prostate Biopsy Cases

PathProfiler analysed 574 prostate biopsy WSI from 61 cases. For the purpose of analysing TAT, one of these 61 cases was excluded as this case consisted of two specimen types: bladder biopsy and prostate biopsy, and data is therefore presented for 60 cases. This case also appeared within the cohort of ‘flagged’ cases and therefore data for the remaining 13 rather than 14 cases, which included the ‘flagged’ WSI, are presented. See Table 3. Data were included for all cases within all cohorts for which there were complete timestamp datapoints; one case within the pre-study cohort had timestamp data missing and was excluded from analysis, but all timestamp data was otherwise available. All comparisons between two times have been assessed using an unpaired *t*-test, level of statistical significance *p* = 0.05.

The mean overall TAT during the study for all prostate biopsy cases was 6.4 days (152.8 h), with mean TAT for non-flagged cases and flagged cases within that cohort being 6.2 days (148.8 h) and 7.0 days (167.4 h), respectively. Whilst the mean TAT of the ‘flagged’ cases is longer than for ‘non-flagged’ cases during the study period, this is <1 day and is not statistically different (difference 18.6 h, *p* = 0.36).

For the purpose of comparing TAT, we have included both a 3-week period from both prior to the study and following the study, both representing periods without PathProfiler in the workflow. Comparing the mean TAT for ‘flagged’ cases during the study with prostate biopsy cases in a period outside the study, there was an apparent significant difference between the study and post-study period (difference of 45.9 h, 95% confidence interval 9.7 to 82.1, *p* = 0.01), but not when compared with the pre-study cohort (difference of 28.1 h, 95% confidence interval −13.1 to 69.4, *p* = 0.18). By comparing with two separate time periods, this serves to illustrate that there is natural variation in TAT, although the reason for the variation is not clear from the data. But this also makes it difficult to compare the impact of PathProfiler on overall TAT during such a short time period. Ideally, such data would be assessed over a longer period with the tool integrated into the workflow in the most efficient manner possible.

The mean Lab TAT is perhaps a better comparator than overall TAT given that this is not impacted by differences in pathologist reporting of cases, which will vary depending upon whether additional work such as immunohistochemistry is required. Therefore, looking specifically at Lab TAT, the mean for the non-flagged cases versus the flagged cases during the study was 2.6 days (64.1 h) versus 2.7 days (64.0 h), which is a non-significant difference of 0.1 h (95% confidence interval −18.6 to 18.6, *p* = 0.99). Comparing the mean Lab TAT for cases in the pre-study cohort of 2.6 days (62.7 h), with that for the flagged cases during the study of 2.7 days (64.0 h), the mean time difference of 1.4 h is also non-significant (95% confidence interval −9.9 to 12, *p* = 0.79), and similarly comparing with the post-study cohort, the mean time difference of 12.1 h is also not significant (95% confidence interval −0.1 to 24.5, *p* = 0.24).

### 3.5. Feedback from the Study Team

The study team met regularly during the study to ensure that any problems were addressed in a timely manner. There were no specific issues raised during this period in relation to the operability of the AI within the workflow and the team felt that the output of the tool was easy to interpret within the LIMS in order that QC issues could be addressed promptly. The subjective impression overall was that the tool was likely most useful in its current form for biopsy specimens rather than resections, but all were keen to roll out the tool amongst more of the specialties to assess its functionality and performance more widely. Formal feedback was obtained from the two lab-based BMSs involved in the study, who both agreed that PathProfiler was helpful in the review of quality of digital images, that the output was easy to visualise, and that on a scale of 1–5 (5 being very likely), they would like to use the tool in the lab again (scores of 4 and 5 out of 5). In terms of impact on TAT that having PathProfiler in the workflow might have, both suspected that there was an impact, one suspecting that TAT might be reduced and the other that it might be increased. Both felt that, having undertaken the study, they had increased awareness of digital image quality in general, PathProfiler had helped their understanding of quality-impacting factors related to WSI and what pathologists found challenging, and that having access to an automated QC tool has the potential to improve digital image quality in the lab in general. General comments related to the challenges with introducing an AI tool for QC, including that there would need to be an overall change in lab process if this were adopted routinely into practice, and that the tool did create additional work in the context of the study through the assessment of some WSI and then consequent remedial action for GS for which there was no genuine quality issue.

## 4. Discussion

In this study, our automated digital image quality tool ‘PathProfiler’ was integrated into the clinical workflow at a point immediately following slide scanning, providing a quantitative measurement of WSI quality by way of a Usability Score. Based on our previous work we set the ‘cut-off’ for the US at 0.4, with WSI scoring 0.4 or less being flagged to the laboratory team in real time. The flagged WSI could therefore be assessed and remedial action instigated, which was typically cleaning and re-scanning of the GS.

A total of 6.6% of WSI from 1254 consecutive GU GS were flagged by PathProfiler, although the majority were from resection-type cases (59%) on which PathProfiler had not been trained. When resections were excluded from the flagged WSI, the figure of 2.7% WSI from non-resection GS (mostly biopsies) reflected more accurately the concurrent subjective assessment of quality of the flagged WSI from resection and non-resection cases. Almost invariably, in the genuine quality-impacted flagged WSI a focus issue was responsible, in line with observations within the literature.

Importantly for consideration for routine practice, when we further analysed the prostate biopsy cases in accordance with the level of severity of the subjective quality issue, we found that for WSI with a severe issue the mean US was 0.2, whereas for minor or non-significant issues it was 0.34 and 0.37, respectively. Whilst this observation is based on small numbers, this is helpful as it would suggest that re-setting the US cut-off from 0.4 to 0.2 or below could potentially improve the specificity in terms of genuinely severely impacted WSI being flagged, which may then reduce the impact on the laboratory team of manual assessment of potentially un-necessarily flagged WSI, which was an issue commented upon in the BMS feedback following the study. For non-resection cases, remedial action was associated both with an objective improvement in the WSI US of re-scanned GS and with a concordant improvement in subjective quality assessment for almost all cases.

Prior to implementing an additional step into the diagnostic pathway, an understanding of the impact of PathProfiler on TAT would also be important. This was a limited 3-week study during which time it is unlikely that a fully efficient process by which to benchmark the time impact of PathProfiler was achieved; however, for prostate biopsy cases during this study, we did not see a significant delay in cases in terms of TAT. This is essential given that TAT are important clinically and are a Key Performance Indicator of the laboratory (and pathologist). Furthermore, the impact on the pathologist of having an optimal quality scan at the time of reporting cannot be underestimated, even if there were observed to be a minor increase in time taken in the lab workflow phase; important in terms of continuity of workflow, reducing frustration, and improving acceptability of DP overall.

Importantly for clinical use, the output of the tool is easy to interpret by users due to the simple colour-coded flag system within the LIS, which was reflected in the feedback from the BMSs involved in the study. The simplicity of the output is an advantage over existing solutions and is relevant in terms of minimising the time needed for assessment and interpretation of the AI output, which could otherwise easily negate some of the positive impact of the solution.

The observations of this feasibility study are significant given that QC of WSI remains a crucial part of the transition to clinical utilisation of DP, and is increasingly recognised as such in commentaries from early adopters of DP [11,14,25,26]. Furthermore, evidence continues to emerge to support intuition that quality WSI are important during both the development of AI tools and in assuring their performance [5,6,26].

In spite of this, there is little evidence regarding the frequency of suboptimal WSI encountered in routine DP clinical practice. Some suggest that around 10% of routine slides could be affected by scanning issues [10]; however, few have systematically assessed this issue. In the real-world setting, quality issues are likely under-reported unless there is a robust and efficient pathway within the workflow for users to flag poor quality WSI for attention; (personal) experience suggests that pathologists tend to mitigate issues typically by GS review, to avoid interruption of their workflow. This is a relatively easy work-around in a DP setting such as ours, where GS are routinely sent out to pathologists concurrently with ‘release’ of the WSI; however, in the alternative setting where GS are not routinely available or a pathologist is working remotely, a re-scan of the GS would be necessary, which may delay the diagnostic process.

A recent study from a large centre with established DP practice reported an overall scan failure rate in a series of over two million slides of 1.19% [27], mostly attributed to machine error such as skipped tissue and failed region of interest detection, with contribution to this figure also from quality issues affecting GS. A similar scan error rate of <1.5% is reported by Ardon et al. [14], with missing tissue and out-of-focus errors accounting together for around 60% of cases. These figures are reassuringly low, but it is unclear from these studies as to the exact parameters used to classify a scan failure. Furthermore, there is likely a degree of subjectivity as to what a pathologist would consider a ‘suboptimal’ WSI that will in part be specimen-dependent and reflective of the degree of scrutiny required for diagnosis. In our own practice, we noted a 2.6% rate of technical failure for digitised WSI during a study on the department-wide DP validation process, which was multifactorial but largely contributed to by focus issues [3]; however, this figure is from the early phases of DP implementation and may not reflect current practice. However, the figure of 2.7% of flagged WSI from non-resection cases in our study cohort would seem therefore to be in keeping with the expected number of suboptimal images in clinical practice, which is reassuring.

### Study Limitations

A limitation of this study is that we have not assessed the ‘false-negative’ rate of PathProfiler. This would potentially have been achievable by concurrently asking GU pathologists during this period to identify WSI of suboptimal quality that had made it past the QC stage and therefore could perhaps be regarded as having been ‘missed’ by PathProfiler; however, we have been mindful of the existing clinical time pressures and did not wish to impact on productivity. Whilst this would be a useful parameter to assess the impact of PathProfiler, the focus of this current study was on the feasibility of using PathProfiler in a clinical workflow. Arguably, any reduction in the number of suboptimal quality WSI slipping through the QC process would be an improvement on the current system.

## 5. Conclusions

We have demonstrated the potential for our automated QC solution ‘PathProfiler’ to be incorporated successfully into the clinical workflow within our digitised pathology laboratory. To the best of the authors’ knowledge, this is the first study of an AI solution for WSI quality within a real-world clinical pathology setting. 

We have shown the capability of the tool to detect and flag WSI of potentially suboptimal quality worthy of manual assessment. The performance of PathProfiler when benchmarked against pathologist subjective quality assessment was most reliable for non-resection type cases, with best concordance seen for prostate biopsy cases, which is unsurprising since PathProfiler was developed in this setting. However, there was proven utility in non-prostate biopsy cases, which is promising and deserving of further investigation. 

Whilst this automated QC tool does not replace the need for careful attention to quality-impacting features during the production of the GS, which will always remain essential, it offers real opportunity for automation of part of the QC process and could also introduce improved consistency in quality through non-reliance on human interpretation. We plan now to investigate the potential utility of PathProfiler in a wider range of specimen types within our laboratory with a view to routine adoption in our workflow, and this may identify areas for further development of the tool to enhance its utility.

Importantly, as we now transition into an era of opportunity for AI in assisting pathologists in diagnosis or adding novel predictive information to the diagnostic assessment, the need for quality WSI upon which such AI assessments can be made is becoming increasingly apparent, and a QC step may not be a feature of such AI technology. It is not yet understood how image quality within a diagnostic setting might impact on AI tools which are currently available to pathologists; however, it might be expected based on observations within a research setting that image quality could impact their reliability. It is foreseeable, then, that a quality tool such as PathProfiler might allow the setting of a minimum quality threshold for a WSI prior to its assessment within an AI pipeline.

## Figures and Tables

**Table 1:** Genitourinary cases analysed by PathProfiler during the study. TURP = transurethral resection of prostate tissue, TUR = transurethral resection specimen (usually of bladder tumour), URE = urology excision/GU resection specimen.

Specimen Type	Number of Cases
Prostate biopsies	61
Prostate TURP	16
Radical prostatectomy (URE)	5
Bladder TUR	23
Bladder biopsies	22
Urethral biopsies	1
Cystectomy (URE)	2
Renal mass biopsy	3
Nephrectomy (URE)	7
Testis biopsies	26
Orchidectomy (URE)	7
Foreskin (URE)	8
Miscellaneous	3
TOTAL CASES	184

**Table 2:** Pathologist subjective assessment of the quality of WSI flagged by PathProfiler with Usability Score of 0.4 or less.

	Nil Significant/Very Minor Issue	Minor Issue	Severe Issue
Total number of slides (n = 83)	41 (49%)	26 (31%)	16 (19%)
Excluding resection cases (n = 34)	2 (6%) (2 bladder TURs)	16 (47%)	16 (47%) (14 prostate biopsies)

**Table 3:** Turnaround time (TAT) for prostate biopsy cases comparing TAT during the study period with that outside the study period. The data presented are for 3 cohorts: the study cohort (divided into the cases which included flagged WSI and non-flagged WSI), and a comparable random 3-week period from prior to the study (comparable cohort 1) and following the study (comparable cohort 2).

	Number of Cases	Mean (sd)	Median (IQR)	Range
Total time from receipt in lab to final authorisation (Overall TAT, hours)				
Study all cases	60	152.8 (64.7)	149.1 (97.3, 183.3)	44.0–333.3
Study non-flagged cases	47	148.8 (63.0)	148.8 (97.1, 176.3)	44.0–333.3
Study flagged cases	13	167.4 (71.5)	150.0 (129.0, 194.4)	44.6–316.5
Comparable cohort 1 (pre-study)	74	139.3 (68.6)	126.0 (101.1, 169)	26.8–366.4
Comparable cohort 2 (post-study *)	57	121.5 (55.9)	120.8 (77.1, 148.9)	24.2–267.4
Total time from receipt in lab to release from lab (Lab TAT, hours)				
Study all cases	60	64.1 (29.5)	50.9 (43.4, 93.7)	22.9–146.0
Study non-flagged cases	47	64.1 (29.3)	50.3 (42.6, 93.8)	22.9–118.2
Study flagged cases	13	64.0 (31.4)	51.6 (45.3, 75.4)	31.0–146.0
Comparable cohort 1 (pre-study)	74	62.7 (31.4)	50.8 (30.5, 97.8)	24.4–114.9
Comparable cohort 2 (post-study *)	57	51.9 (33.3)	32.2 (27.6, 89.9)	22.8–128.4
Total time from release from lab to pathologist authorisation (Pathologist TAT, hours)				
Study all cases	60	88.7 (57.4)	94.7 (46.4, 127.2)	2.0–240.2
Study non-flagged	47	84.6 (56.5)	72.8 (45.8, 126.1)	3.4–240.2
Study flagged	13	103.4 (60.3)	95.6 (77.5, 151.2)	2.0–191.1
Comparable cohort 1 (pre-study)	74	76.6 (63.3)	69.8 (25.5, 119.6)	1.9–337.3
Comparable cohort 2 (post-study *)	57	69.6 (56.2)	51.3 (22.2, 116.3)	1.4–192.3

* Note that the post-study laboratory workflow was the same as pre-study; neither comparable period (pre- or post-study) included PathProfiler in the workflow. WSI = whole slide images, sd = standard deviation, IQR = interquartile range.

## Data Availability

The datasets used and/or analysed during the current study are available from the corresponding author on reasonable request.
